# Induced pluripotent stem cell-derived limbal epithelial cells (LiPSC) as a cellular alternative for *in vitro* ocular toxicity testing

**DOI:** 10.1371/journal.pone.0179913

**Published:** 2017-06-22

**Authors:** Edith Aberdam, Isabelle Petit, Linda Sangari, Daniel Aberdam

**Affiliations:** INSERM U976 and Université Paris-Diderot, Hôpital St-Louis, Paris, France; Cedars-Sinai Medical Center, UNITED STATES

## Abstract

Induced pluripotent stem cells hold great potential to produce unlimited amount of differentiated cells as cellular source for regenerative medicine but also for *in vitro* drug screening and cytotoxicity tests. Ocular toxicity testing is mandatory to evaluate the risks of drugs and cosmetic products before their application to human patients by preventing eye irritation or insult. Since the global ban to use animals, many human-derived alternatives have been proposed, from *ex-vivo* enucleated postmortem cornea, primary corneal cell culture and immortalized corneal epithelial cell lines. All of them share limitations for their routine use. Using an improved protocol, we derived limbal epithelial cells from human induced pluripotent stem cells, named LiPSC, that are able to be passaged and differentiate further into corneal epithelial cells. Comparative RT-qPCR, immunofluorescence staining, flow cytometry analysis and zymography assays demonstrate that LiPSC are morphologically and molecularly similar to the adult stem cells. Moreover, contrary to HCE, LiPSC and primary limbal cells display similarly sensitive to cytotoxicity treatment among passages. Our data strongly suggest that LiPSC could become a powerful alternative cellular model for cosmetic and drug tests.

## Introduction

Ocular toxicity testing is mandatory to evaluate the risks of drugs and cosmetic products before their application to human patients. Moreover, all manufactured consumer products and their ingredients must be tested for potential eye irritation to assure the public of their safety. Since 1940, the international gold standard assay for acute ocular toxicity is the rabbit *in vivo* Draize eye test [1]. This test has the advantage to evaluate drug toxicity *in vivo* in a physiology context that includes immune system, endothelial and neural cells. However, there are major criticisms for the use of this method: ethical issue of animal suffering, anatomical, structural, physiological and biochemical differences between the human and the rabbit eye, as well as time and cost-consuming. In addition, the Draize test displays a poor reproducibility among laboratories. Although still widely used, efforts have been made to identify alternative non-animal methods to test potential irritant effect of chemicals [2]. Ex-vivo cornea, although of low availability, could be cultivated from surgical waste but for short time, limiting their routine use. The corneal epithelium on the front surface of the eye is renewed constantly by limbal epithelial stem cells (LEC) that reside at the corneo-scleral junction, known as the limbus. Contrary to corneal epithelial cells (CEC), LEC lack expression of differentiation markers such as cytokeratins 3 and 12 [3,4]. However, because they undergo rapid replicative senescence *in vitro*, primary cultivation of limbal or corneal epithelial cells cannot be used routinely as an alternative cellular model for ocular toxicity. A new cellular model called LabCyte CORNEA-MODEL is provided by a Japan company (Tissue Engineering Co, Ltd). This model is made of primary differentiated human corneal epithelial cells isolated from a US eye bank. Although a good predictor of in vivo toxicity [5], it remains limited by the great variability between post-mortem samples. Alternatively, the commercially available EpiOcular cell culture model (MatTek) has been used in toxicity and irritation tests [6]. However, it consists of human epidermal cells and not corneal cells.

The recent development of immortalized corneal epithelial cell lines has opened the possibility to overcome replicative senescence. The HCE cell line model, made of immortalized epithelial cells by SV40 is provided by SkinEthic and used for ocular toxicity studies as monolayer studies [7] and 3D models [8]. However, this cell line expresses, in addition to corneal markers found in vivo, markers of simple epithelia [5]. Therefore, there is still the need to develop a cellular model closely resembling limbal and corneal epithelial cells from an unlimited source, with little donor variability [9].

Human pluripotent stem cells (PSC) display both unlimited growth capacity and ability to differentiate into any cell type [10]. Several groups, including ours, have recently developed methods for preferential differentiation of PSC into CEC [11–15]. The resulting cells express corneal epithelial markers but also K3/K12, suggesting that the cells derived from PSC in all these studies represent mainly progenitor CEC and not LEC. In one recent study, a comparative proteomic analysis revealed that the corneal cells derived from PSC represent an intermediate state between LEC and CEC [16].

Here, we thoroughly modified the culture condition to produce a propagatable pure population of iPSC-derived LEC (LiPSC), closely similar to somatic LEC but still able to terminally differentiate at confluency in presence of calcium. Finally, we show that, among passages, LiPSC behave similarly to primary LEC upon *in vitro* toxicity test.

## Materials and methods

### LESC isolation and amplification

Cadaveric limbal tissue composed of peripheral cornea and limbus were obtained from the Fondation Ophtalmologique Alphonse de Rothschild (Paris, France); written informed consent for research had been obtained. To isolate LEC, peripheral cornea were incubated in 0.5% dispase II (Roche) overnight at 4°C. The epithelial sheet was separated from the stroma with fine forceps and placed in 0.05% trypsin/0.01% EDTA (Gibco) for 20 minutes at 37°C with gentle shaking. The suspended cells were collected and plated on 60 Gy irradiated- Swiss-3T3 fibroblast feeder layer in DMEM/Ham’s F12 at 1:1 ratio, supplemented with 5 μg/ml human insulin (Sigma), 0.5 μg/ml hydrocortisone (Sigma), 2 nM triiodothyronine (Sigma), 0.1 nM cholera toxin (Sigma), 10 ng/ml human recombinant EGF(Life Technologies), 10 μM ROCK inhibitor (Y27632, Euromedex) and 5% FCS (FCII, Hyclone).

Alternatively, cells were isolated and cultivated in defined medium (Epilife, Thermo Fisher) Cells in passages 2 to 4 from different donors were used in our experiments.

### Cells and limbal differentiation

**Fig 1:**
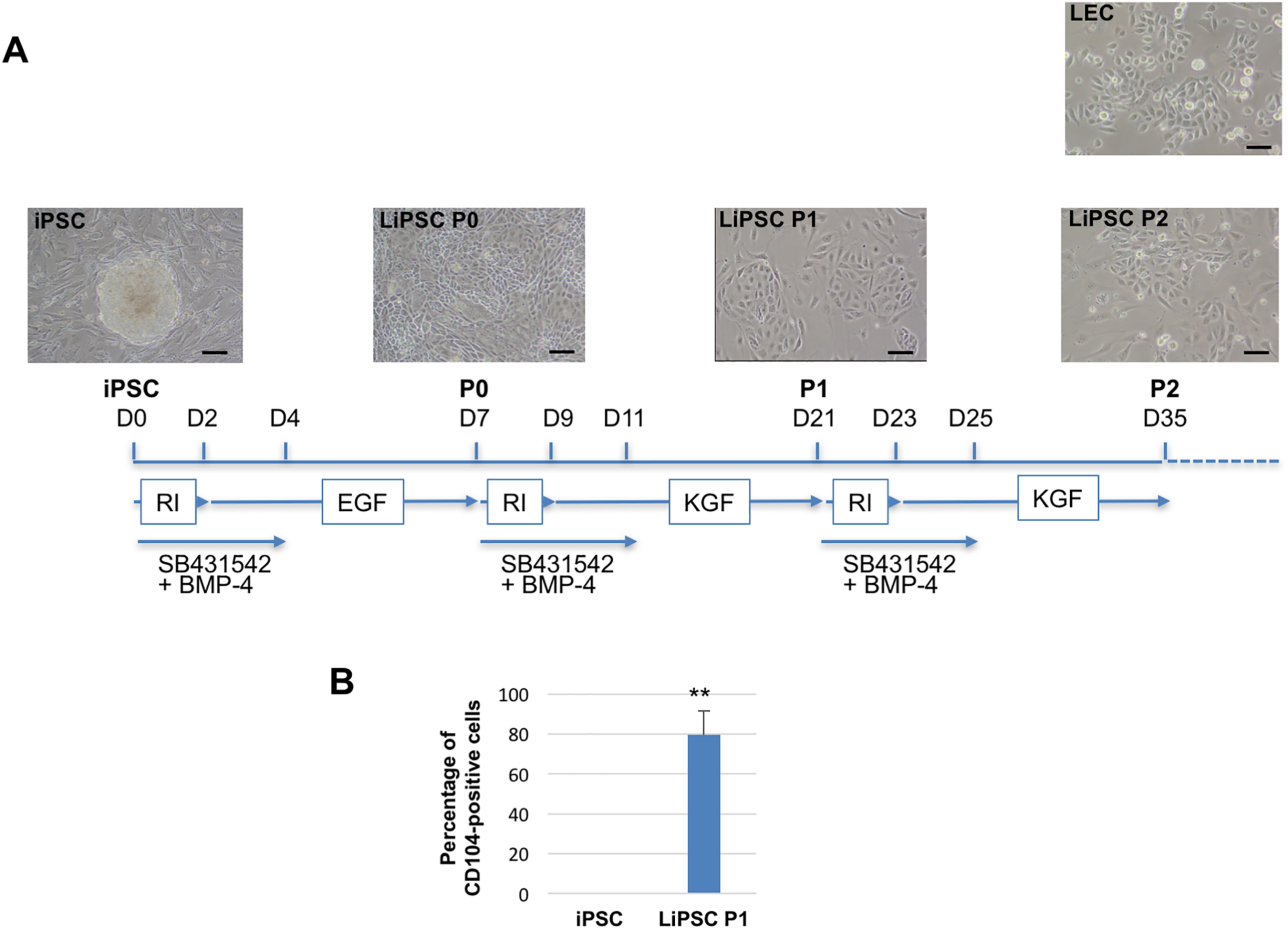
Production of limbal-like cells (LiPSC) from human iPSC. (**A**) Schematic figure displaying a multistep protocol of differentiating human iPSC to limbal-like cells. Time of treatment is illustrated by horizontal arrays. ROCK inhibitor (RI); TGF-beta inhibitor (SB431542). Morphology of the cells at each stage is illustrated at the top and compared to LEC (right). Scale bar, 50 μm. (**B**) Flow cytometry analysis of undifferentiated iPSC and LiPSC at P1 with anti-CD104 antibody. n = 3. **P <0.01 statistically significant by student's *t*-test.

The experimental design of this study is schematically described in Fig 1A. Four sources of human iPSC were used in this study and displayed similar behavior. AnaW04 line has been previously obtained from hair follicle keratinocyte reprogramming [17], iPSC-DFC was described previously [18], iPSC-B5CRE was obtained from A. Bennaceur-Griscelli (Paris) and iPSC-29.3 line from H. Zhou (Nijmegen). The last three were derived from human dermal fibroblasts. Undifferentiated iPSC were differentiated according to our published protocol [11] that was modified here as follow. Briefly, irradiated fibroblasts isolated from peripheral cornea (pCOF), were seeded on 0.8 mg/ml collagen IV (Sigma)-coated dishes. Then, limbal commitment was induced by seeding iPSC (1:6) in DMEM (Gibco), Ham’s F12 (Gibco) (2:1), supplemented with 10% fetal bovine serum FCII (Hyclone), 2 mM glutamine (Gibco), 1 mM Sodium Pyruvate (Gibco), 100 U/ml Penicillin/100 μg/ml Streptomycin (Gibco), 0.2 mM Adenine (Sigma), 5 μg/ml human Insulin (Sigma), 0.5 μg/ml Hydrocortisone (Sigma), 2 nM Tri-iodothyronine (Sigma), 0.1 nM Cholera Toxin (Sigma) (Epithelial medium) for one week (LiPSC, P0) supplemented as described in Fig 1A. Cells were then dissociated by Accutase and seeded (10,000 cells/cm^2^) on 0.8 mg/ml collagen IV-coated dishes on 3T3-J2 irradiated feeder, in DMEM (Gibco), Ham’s F12 (Gibco) (1:1), supplemented with 4% fetal bovine serum FCII (Hyclone), 2 mM glutamine (Gibco), 1 mM Sodium Pyruvate (Gibco), 100 U/ml Penicillin/100 μg/ml Streptomycin (Gibco), 5 μg/ml Insulin (Sigma), 0.5 μg/ml Hydrocortisone (Sigma), 2 nM Tri-iodothyronine (Sigma), 0.1 nM Cholera Toxin (Sigma) (limbal medium) supplemented as described in Fig 1 for 10 to 15 days (LiPSC, P1). LiPSC at P2 were obtained as described for LiPSC P1. When indicated, 10 μM of SB431542 (Euromedex), 25 ng/ml BMP-4 (R&D), 5 ng/ml KGF (Life Technology), 5 ng/ml EGF (Life Technology) and 10 μM ROCK inhibitor Y-27632 (Euromedex) were added.

### Antibodies and immunofluorescence staining

The following antibodies were used for immunofluorescence staining: rabbit polyclonal anti-K14 (Covance, Biolegend, 1/400), mouse monoclonal anti-K3 AE5 (Millipore, 1/100), mouse monoclonal anti-P63 4A4 (Abcam, 1/50), rabbit polyclonal anti-Pax6 (Millipore, 1/300e), mouse monoclonal anti- E-cadherin (R&D system, 1/100e), donkey anti-mouse or goat anti-rabbit secondary antibodies conjugated with Alexa 594 or 488 (1/5000, Invitrogen). Cells were fixed in 4% paraformaldehyde, washed in PBS and permeabilized with 0.5% Triton X-100. Primary and secondary antibodies were diluted in PBS containing 3% BSA and incubated with samples at room temperature for 1 hour. Between all steps, cells were washed in PBS 3x10 min. Stained cells were mounted using Dapi Fluoromount-G (SouthernBiotech) and examined under a Nikon Eclipse Ti fluorescence microscope equipped with an OrcaFlash 4.0 LT camera (Hamamatsu). The software used for analysis was NIS-Elements.

### Flow cytometric analysis

Differentiated hiPSC were dissociated by Accutase, then fixed and permeabilized with eBioscience Foxp3 fixation/perm kit (Invitrogen) according to manufacturer instructions. The cells were then incubated for 30 min at 4°C with a mouse monoclonal anti-K14 (1/500, Millipore), Alexa fluor 488 mouse anti-human Pax6 (1/20, BD) and anti-E-cadherin (1/100, R&D) antibodies diluted in 0.1% bovine serum albumin (BSA, Sigma) in PBS. As control for Pax6 staining, Alexa Fluor 488 IgG2a, (1/5, BD) was used. Then the cells were washed with 2% FBS in PBS(-) and incubated for 30 min at 4°C with Alexa Fluor 488 donkey anti-mouse antibody (1/700, Life Technologies) as secondary antibody for E-cadherin and K14. The cells were then rinsed twice with 2% FBS in PBS. Cells were labelled with FITC-anti human CD104 (Biolegend, 1/20) without fixation/permeabilization steps. Fluorescence intensities were analyzed on a FC 500 cytometer (Beckman coulter) flow cytometer with CXP software.

### RNA extraction and RT-qPCR

RNA was extracted using the RNeasy minikit (Qiagen) and cDNA were synthetized from 1 μg RNA using the iScript cDNA synthesis kit (Bio-Rad). qRTPCR was performed in triplicate using the 2XSYBR Green qPCR master mix (Biotools) according to the manufacturer’s protocol in the Light Cycler 480 II (Roche). Expression of each genes was calculated by the 2^ΔΔCt^ method. The value of each reaction was normalized to GUSB (b-glucuronidase) as control.

Primers were as follow:

K14 F: TTCTGAACGAGATGCGTGAC; K14 R: GCAGCTCAATCTCCAGGTTC

ΔNP63 F: GCCAGGGTAAGGGGTAAAAG; ΔNP63 R: CCCAAAAGCCCATAACAGAA

Pax6 F: TCCTTCTCGCTGGCTGTAAT; Pax6 R: CCTGGAGCTCTGTTTGGAAG

Ecad F: CCCACCACGTACAAGGGTC; Ecad R: CTGGGGTATTGGGGGCATC

GUSB F AGAGTGGTGCTGAGGATTGG; GUSB R: CCCTCATGCTCTAGCGTGTC

### Cytotoxicity test

CellTiter 96^®^ Non radioactive Cell proliferation Assay (MTT, Promega) was performed on LEC, LiPSC and HCE. Cells were incubated with 0 to 0.01% SDS for 48H or with 0 to 0.004% Benzalkonium for 10 minutes, washed with PBS and cultivated for 48H in appropriate culture medium before performing test according to the manufacturer’s protocol. Results are expressed in percentage of cellular viability as compared to the non-treated control condition.

### MMP9 detection

Relative MMP9 secretion was quantified from conditioned media of the different cell lines by Quantinine ELISA kit (R&D Systems). The culture medium was collected and centrifuged at 14,000 rpm for 5 minutes at 4°C to remove cell debris. For zymography detection of MMP-9 activity, the supernatant was mixed with 5× non-reducing sample buffer (4:1, vol/vol) and electrophorezed on a 10% SDS-polyacrylamide gel containing 0.1% gelatin as a substrate for MMP-9. After electrophoresis, gels were washed in 3% Triton X-100 for 1 hour to remove SDS and then incubated for 16 hours at 25°C in developing buffer (50 mM Tris, 40 mM HCl, 200 mM NaCl, 5 mM CaCl_2_, and 0.2% Brij) on a rotary shaker. After incubation, gels were stained in 30% methanol, 10% acetic acid, and 0.5% (wt/vol) Coomassie brilliant blue for 1 hour followed by destaining.

## Results and discussion

**Fig 2:**
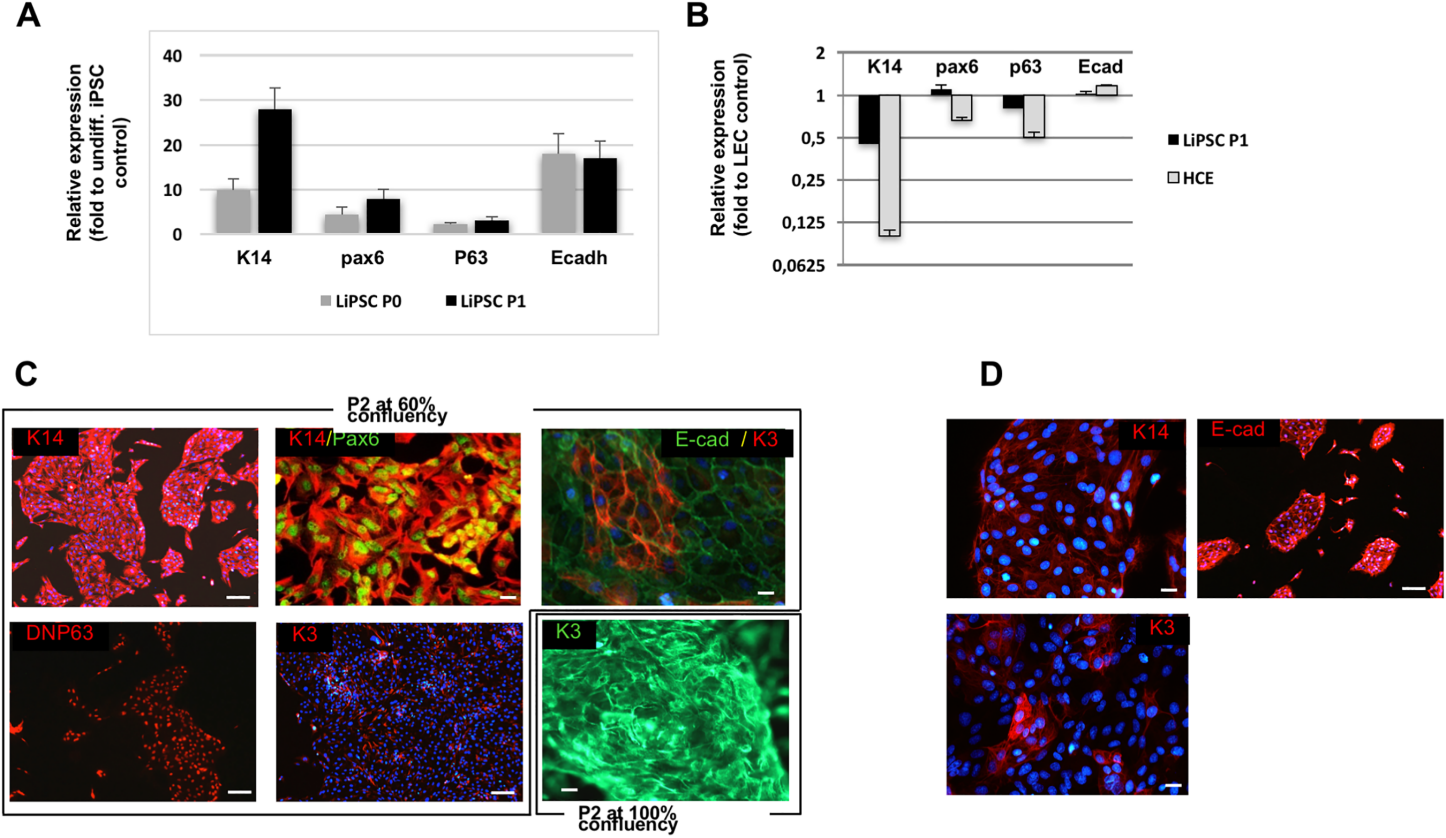
LiPSC express limbal-specific markers. (**A-B**) Reverse transcriptase-quantitative PCR (RT-qPCR) on the expression of limbal-specific genes in LiPSC at P0 and P1 (**A**) and in LiPSC at P1 or HCE (**B**). The relative expression of each transcript was calculated as a fold-change relative to undifferentiated iPSC (**A**) or LEC (**B**). The figures A and B represent the mean of 4 independent biological samples, performed in triplicate. (**C**) Representative immunofluorescent staining of LiPSC at P2 for the expression of K14, Pax6, P63, E-Cadherin and K3 at 60% confluency or 100% confluency (bottom right). (**D**) P2 cells after thawing were immunostained with K14, E-cadherin and K3. Hoechst is shown in blue. Scale bar, 100 μm for all pictures.

Major modifications were introduced to our published protocol [12] to obtain homogenous and expandable cell population of LiPSC from human iPSC (Fig 1A). To initiate limbal commitment, iPSC were cultivated in a limbal-like niche environment. For that purpose, conditioned medium from mitomycined corneal stromal cells [12] was replaced by a feeder made of irradiated limbal stromal cells (pCOF). It is well known that inhibition of TGF-ß upregulates E-cadherin expression [19]. Addition of 10μM SB431542 (inhibitor of TGF-ß) from day 0 to day 4 at each passage enhanced and maintained expression of E-cadherin for proper adherent junctions (Fig 2A and 2C). From P1 and beyond, LiPSC were cultured according to Miyashita and co-authors that have recently designed a protocol for better maintenance of somatic LESC for few months *in vitro* [20]. Therefore, the serum concentration was reduced to 4%, the medium DMEM/F12 modified to ratio 1:1, adenine was removed and EGF was replaced by keratinocyte growth factor (KGF) a paracrine factor that maintains the proliferation and survival of LEC through the specific induction of p63, a master transcription factor of these epithelial cells [20,21]. In addition, Y-27632, the cell survival ROCK inhibitor, was systematically added at each passage for two days while EGF or KGF was omitted at this time. The use of Accutase (Sigma) instead of trypsin to detach intact the small epithelial colonies at each passage greatly improved the survival of LiPSC.

This modified protocol improved limbal commitment that allowed LiPSC propagation for a few passages. LiPSC became homogenous morphologically over passages and similar to primary LESC (Fig 1A, right). The cell surface marker CD104 (integrin beta4 receptor) is specifically expressed on limbal epithelial cells and appears during limbal commitment of iPSC. At P1, more than 80% LiPSC are positive for integrin beta4, as revealed by flow cytometry (Fig 1B). Relative expression of limbal-specific genes was analyzed by RT-qPCR between undifferentiated iPSC and LiPSC. As soon as P0, cytokeratin K14, Pax6, P63 and and E-Cadherin all genes specifically expressed by LEC, were activated as compared to undifferentiated iPSC and further increased at P1 (Fig 2A). When compared to LEC and HCE, expression of these genes in LiPSC was closer to LEC than to HCE, attesting that our differentiation protocol could be a good alternative to HCE model (Fig 2B). In Fig 2C, immunofluorescent staining analysis confirmed that LiPSC are positive for K14, Pax6, P63 and E-cadherin (Fig 2A) but almost negative for K3, marker of differentiated CEC. As expected for LEC-like cells under differentiation conditions (addition of 1.5 mM calcium on confluent cells for 5 days), LiPSC started to stratify and displayed K3 (Fig 2C, right). After frozen/thaw cycles, LiPSC at P2 displayed expression of E-Cadherin, K14 and few cells expressed K3 (Fig 2D).

**Fig 3:**
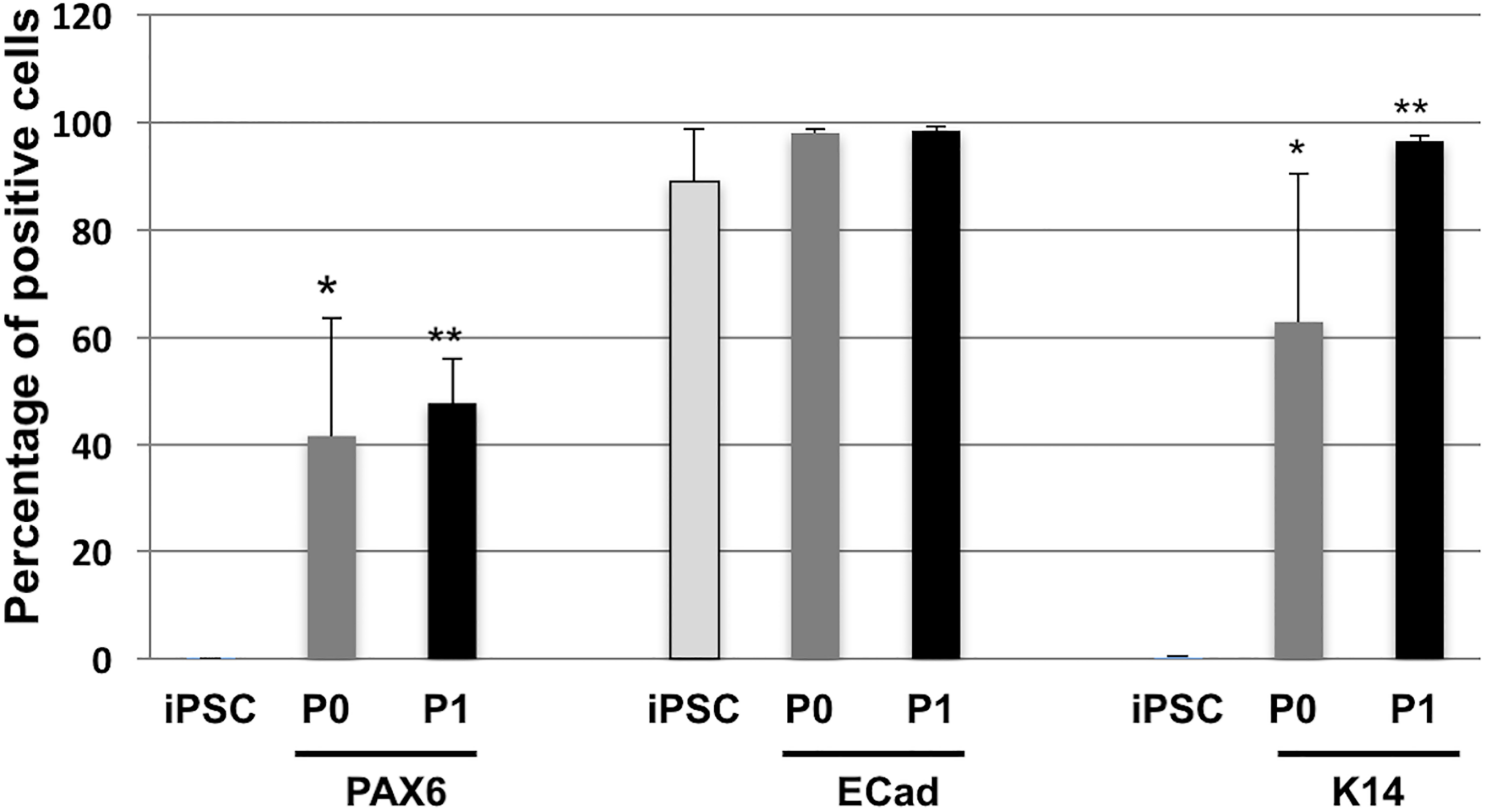
LiPSC at P1 are homogenous cell population. Percentages of Pax6, K14 and E-cadherin positive cells in undifferentiated iPSC, LiPSC at P0 and P1 were determined by bar chart of flow cytometry analysis from 5 independent experiments, *P <0.001, **P <8E^-7^ statistically significant by student's *t*-test.

**Fig 4:**
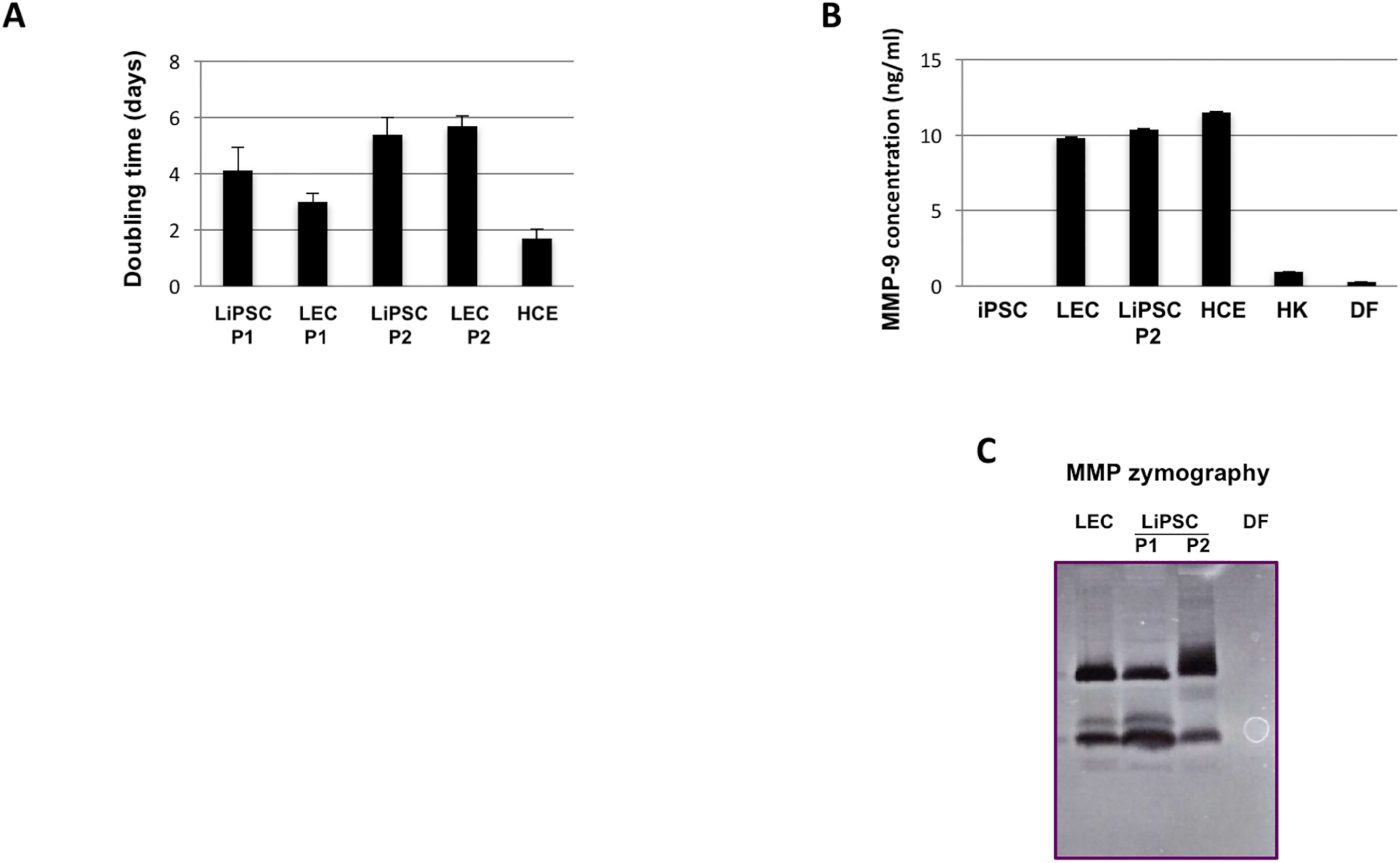
LiPSC behave like LEC. **(A**). Cell numbering was used from 3 independent experiments to calculate the doubling time rate between LEC, LiPSC and HCE. (**B,C**). MMP-9 content was analyzed from conditioned medium of different cell types. ELISA (B) quantified its relative amount (ng/ml) and its activity was analyzed by zymography (**C**). HK: primary human keratinocytes; DF: primary human dermal fibroblasts.

To quantify the proportion of limbal-like cells at P0 and P1, flow cytometry was done with limbal-specific markers and compared to undifferentiated iPSC (Fig 3). LiPSC at P1 were positive for Pax6, K14 and E-cadherin. It further demonstrates that the LiPSC at P1 is a homogenous population of limbal-like cells, still able to spontaneously differentiate into K3^+^-corneal cells. It should be noticed that the representation of Pax6-positive LiPSC by flow cytometry analysis was lower than that observed by immunofluorescence staining (Fig 2C). This feature was also observed for LEC (data not shown). Doubling time of LiPSC was similar to LEC, slightly increasing between P1 to P2 while the SV40-immortalized HCE proliferated much faster with a lower doubling time (Fig 4A). Matrix metalloproteinase-9 (MMP-9) plays an important role in the outgrowth and survival of expanded human LESC [22]. LiPSC produced and secreted similar amount of MMP-9 to LEC as revealed by Elisa (Fig 4B) and zymography assays (Fig 4C), respectively. All these data strongly suggest that LiPSC became LEC-like cells. Similar results were obtained with 3 other iPSC lines (data not shown) A preliminary comparative transcriptome analysis of LiPSC at P0 to P2 versus adult human LEC has been performed from 2 independent biological samples (data not shown). A vast majority of genes (30,213 coding known transcripts) were similarly expressed in LiPSC and LEC. At P2, 667 were expressed only in LEC and 464 solely in LiPSC.

**Fig 5:**
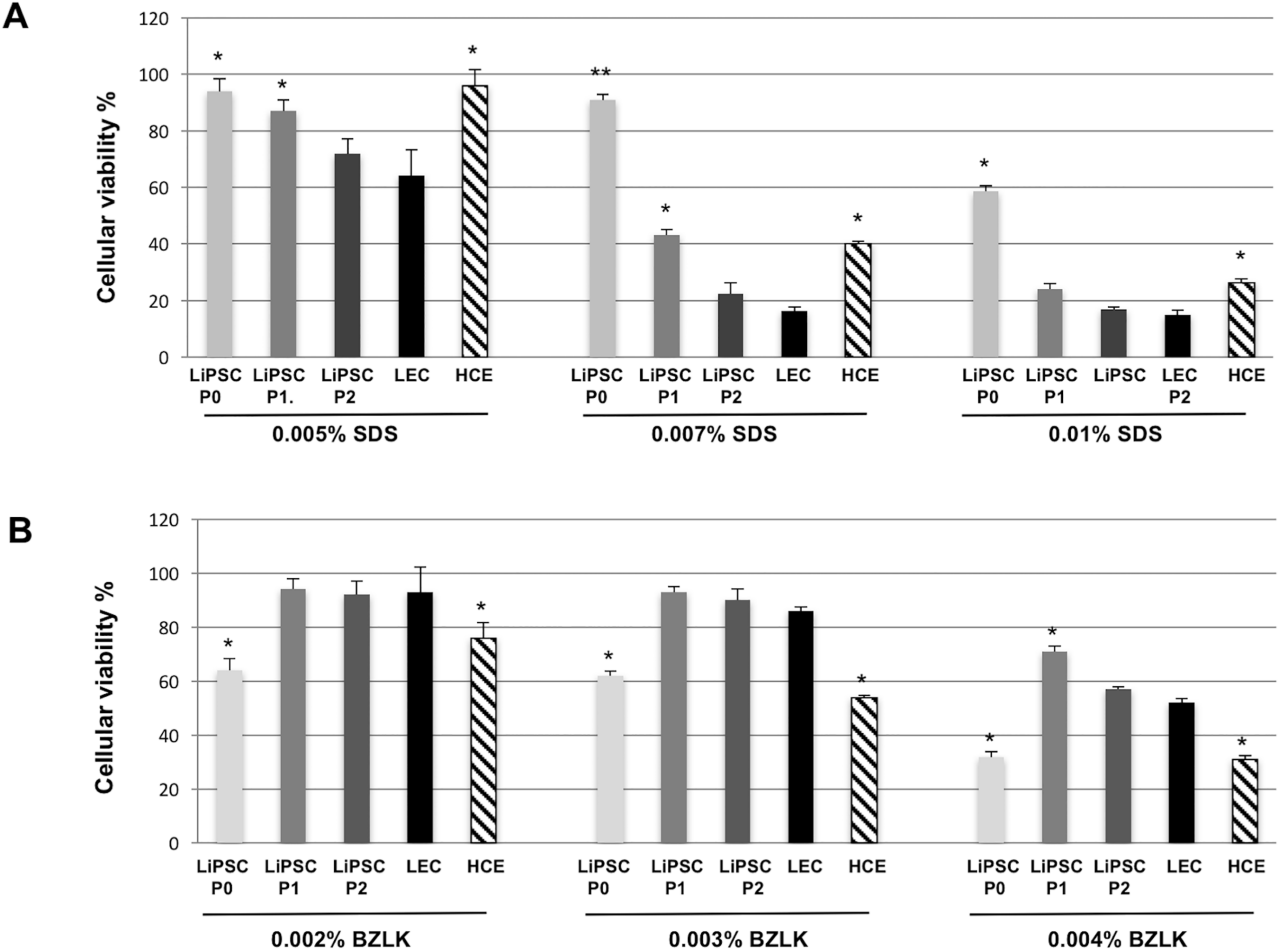
Cytotoxicity test for LiPSC as compared to LEC and HCE. LEC, LiPSC (P0 to P2) and HCE were treated for 48H with SDS **(A**) or with benzalkonium (**B)** at different concentrations, as detailed in Methods section. Cell viability was calculated as compared to untreated cells in six replicate. *P <0.05, **P <0.01 statistically significant by student's *t*-test. n = 6.

To evaluate the potential of LiPSC as an alternative ocular toxicity model, LiPSC were treated at passage P0 to P2 with increasing amounts of SDS and Benzalkonium (BZLK) (Fig 5). SDS is widely used in cosmetology for its surfactant properties. The quaternary ammonium BZLK is a component used to stabilize cosmetic and pharmacologic products, including eye drops. Cell viability of treated LiPSC was compared to LEC and HCE. At P0, LiPSC were systematically more resistant to SDS toxicity than LEC and even to HCE (Fig 5A). However, remarkably, this behavior changed with passage since LiPSC at P2 became identical to LESC and thus closer to physiology than HCE. Similarly, upon BZLK treatment, LiPSC at P2 behaved more like LEC than HCE (Fig 5B). Of note, HCE are more resistant to SDS toxicity but less to BZLK than LEC, making LiPSC more reliable than HCE for cytotoxicity test. This feature was also observed when cells were treated with TritonX-100 between 0.1 to 1% (data not shown). DMSO and glycerol were used as negative controls and did not induce toxicity to LEC or LiPSC (not shown).

In conclusion, the present study demonstrates for the first time that LiPSC and LESC are morphologically and molecularly similar. LiPSC could become an efficient, reproducible and physiological cellular model alternative to the SV40-immortalized human corneal epithelial cell HCE line, routinely used by cosmetologic industries. Moreover, it could alleviate the use of animals to test therapeutic drugs for ocular toxicity before clinical trials, even though *in vivo* environment (including interactions between endothelial, neural and immune cells) cannot be mimicked by in vitro models. Of relevance for their potential use as toxicity cellular model, LiPSC (at passage 1 or 2) can be frozen and thawed, while retaining their limbal phenotype (Fig 2D). The significant challenge for commercialization of such cells is the ability to consistently produce both starting material iPSCs and the differentiated cells in the quantity, quality, and purity required by the pharmaceutical industry.
